# Supplementary material for: Multi‐dataset identification of innovative feature genes and molecular mechanisms in keratoconus
Source: J Cell Mol Med. 2024 Sep 19;28(18):e70079. doi: 10.1111/jcmm.70079 (PMC11412914; doi:10.1111/jcmm.70079)
Supplement: Supplementary file 2 — Material S2. [file JCMM-28-e70079-s002.zip › 7.GSEA analysis/3.DBNDD1/KEGG_METABOLISM_OF_XENOBIOTICS_BY_CYTOCHROME_P450.html]

Details for gene set KEGG\_METABOLISM\_OF\_XENOBIOTICS\_BY\_CYTOCHROME\_P450[GSEA]

|  || Dataset | DBNDD1.group.cls#h\_versus\_l |
| Phenotype | group.cls#h\_versus\_l |
| Upregulated in class | l |
| GeneSet | KEGG\_METABOLISM\_OF\_XENOBIOTICS\_BY\_CYTOCHROME\_P450 |
| Enrichment Score (ES) | -0.36960635 |
| Normalized Enrichment Score (NES) | -1.1696492 |
| Nominal p-value | 0.2319688 |
| FDR q-value | 0.69015384 |
| FWER p-Value | 0.993 |
Table: GSEA Results Summary

  

Fig 1: Enrichment plot: KEGG\_METABOLISM\_OF\_XENOBIOTICS\_BY\_CYTOCHROME\_P450      
 Profile of the Running ES Score & Positions of GeneSet Members on the Rank Ordered List

  

| SYMBOL | TITLE | RANK IN GENE LIST | RANK METRIC SCORE | RUNNING ES | CORE ENRICHMENT || 1 | UGT1A8 | na | 313 | 0.502 | 0.0438 | No |
| 2 | UGT2B11 | na | 1393 | 0.345 | 0.0489 | No |
| 3 | UGT2B4 | na | 2640 | 0.266 | 0.0409 | No |
| 4 | UGT1A6 | na | 2660 | 0.265 | 0.0683 | No |
| 5 | CYP2E1 | na | 3730 | 0.223 | 0.0608 | No |
| 6 | ADH6 | na | 3734 | 0.222 | 0.0841 | No |
| 7 | DHDH | na | 4238 | 0.205 | 0.0912 | No |
| 8 | CYP2C18 | na | 4789 | 0.187 | 0.0949 | No |
| 9 | ADH1B | na | 5723 | 0.158 | 0.0846 | No |
| 10 | UGT2B28 | na | 6027 | 0.150 | 0.0916 | No |
| 11 | GSTM5 | na | 7233 | 0.119 | 0.0694 | No |
| 12 | CYP2C9 | na | 7646 | 0.111 | 0.0692 | No |
| 13 | GSTT1 | na | 7801 | 0.107 | 0.0760 | No |
| 14 | ALDH3B1 | na | 8402 | 0.096 | 0.0688 | No |
| 15 | CYP3A4 | na | 8583 | 0.093 | 0.0734 | No |
| 16 | GSTA5 | na | 9536 | 0.078 | 0.0541 | No |
| 17 | GSTZ1 | na | 9544 | 0.078 | 0.0620 | No |
| 18 | GSTO2 | na | 9799 | 0.074 | 0.0625 | No |
| 19 | CYP2C8 | na | 10615 | 0.063 | 0.0456 | No |
| 20 | UGT2A3 | na | 13213 | 0.036 | -0.0255 | No |
| 21 | CYP2B6 | na | 14096 | 0.029 | -0.0478 | No |
| 22 | MGST2 | na | 14614 | 0.026 | -0.0601 | No |
| 23 | MGST3 | na | 14793 | 0.024 | -0.0626 | No |
| 24 | GSTP1 | na | 17472 | 0.006 | -0.1392 | No |
| 25 | UGT2B10 | na | 17872 | 0.004 | -0.1503 | No |
| 26 | ADH7 | na | 17985 | 0.003 | -0.1533 | No |
| 27 | CYP2C19 | na | 18485 | -0.001 | -0.1676 | No |
| 28 | GSTO1 | na | 18920 | -0.004 | -0.1797 | No |
| 29 | UGT2B15 | na | 18962 | -0.004 | -0.1804 | No |
| 30 | GSTK1 | na | 19412 | -0.008 | -0.1926 | No |
| 31 | ADH5 | na | 20394 | -0.016 | -0.2191 | No |
| 32 | ALDH3A1 | na | 23496 | -0.049 | -0.3034 | No |
| 33 | UGT2B7 | na | 24690 | -0.066 | -0.3308 | No |
| 34 | EPHX1 | na | 24922 | -0.070 | -0.3302 | No |
| 35 | GSTA4 | na | 25661 | -0.081 | -0.3429 | No |
| 36 | CYP3A7 | na | 26069 | -0.089 | -0.3453 | No |
| 37 | AKR1C4 | na | 26497 | -0.098 | -0.3474 | No |
| 38 | GSTM3 | na | 27131 | -0.111 | -0.3540 | No |
| 39 | CYP1A2 | na | 27673 | -0.123 | -0.3567 | Yes |
| 40 | GSTT2 | na | 28006 | -0.131 | -0.3525 | Yes |
| 41 | UGT2A1 | na | 28049 | -0.132 | -0.3398 | Yes |
| 42 | ALDH3B2 | na | 28095 | -0.133 | -0.3271 | Yes |
| 43 | GSTA2 | na | 28419 | -0.141 | -0.3216 | Yes |
| 44 | GSTA3 | na | 28451 | -0.142 | -0.3075 | Yes |
| 45 | AKR1C1 | na | 28597 | -0.146 | -0.2964 | Yes |
| 46 | ADH1A | na | 29956 | -0.187 | -0.3158 | Yes |
| 47 | MGST1 | na | 30100 | -0.191 | -0.2999 | Yes |
| 48 | AKR1C3 | na | 31537 | -0.242 | -0.3159 | Yes |
| 49 | CYP3A43 | na | 31748 | -0.251 | -0.2956 | Yes |
| 50 | GSTM2 | na | 31785 | -0.253 | -0.2700 | Yes |
| 51 | CYP3A5 | na | 31787 | -0.253 | -0.2434 | Yes |
| 52 | ALDH1A3 | na | 31867 | -0.257 | -0.2187 | Yes |
| 53 | GSTM1 | na | 32158 | -0.273 | -0.1983 | Yes |
| 54 | CYP2F1 | na | 32367 | -0.286 | -0.1743 | Yes |
| 55 | ADH4 | na | 32701 | -0.306 | -0.1517 | Yes |
| 56 | CYP2S1 | na | 33107 | -0.337 | -0.1280 | Yes |
| 57 | ADH1C | na | 33609 | -0.380 | -0.1024 | Yes |
| 58 | GSTM4 | na | 33662 | -0.386 | -0.0634 | Yes |
| 59 | CYP1B1 | na | 33997 | -0.429 | -0.0279 | Yes |
| 60 | CYP1A1 | na | 34212 | -0.466 | 0.0149 | Yes |
Table: GSEA details [plain text format]

  

Fig 2: KEGG\_METABOLISM\_OF\_XENOBIOTICS\_BY\_CYTOCHROME\_P450      
 Blue-Pink O' Gram in the Space of the Analyzed GeneSet

  

Fig 3: KEGG\_METABOLISM\_OF\_XENOBIOTICS\_BY\_CYTOCHROME\_P450: Random ES distribution      
 Gene set null distribution of ES for **KEGG\_METABOLISM\_OF\_XENOBIOTICS\_BY\_CYTOCHROME\_P450**
